# Eucommia granules activate Wnt/β-catenin pathway, and improve oxidative stress, inflammation, and endothelial injury in preeclampsia rats

**DOI:** 10.1590/acb391524

**Published:** 2024-04-15

**Authors:** Xia Huang, Guangyang Xing, Cui Zhang, Xiaotong Sun

**Affiliations:** 1 Gansu Provincial Hospital – Department of Gynecology and Obstetrics – Lanzhou, China.; 2 Gansu University of Chinese Medicine – Affiliated Hospital – Department of Pathology – Lanzhou, China.

**Keywords:** Pre-Eclampsia, Oxidative Stress, Inflammation, Vascular Injury, Wnt Signaling Pathway

## Abstract

**Purpose:**

Pre-eclampsia (PE) is a pregnancy-related complication. Eucommia is effective in the treatment of hypertensive disorders in pregnancy, but the specific effects and possible mechanisms of Eucommia granules (EG) in PE remain unknown. The aim of this study was to investigate the effects and possible mechanisms of EG in PE rats.

**Methods:**

Pregnant Sprague Dawley rats were divided into five groups (n = 6): the control group, the model group, the low-dose group, the medium-dose group, and the high-dose group of EG. The PE model was established by subcutaneous injection of levonitroarginine methyl ester. Saline was given to the blank and model groups, and the Eucommia granules were given by gavage to the remaining groups. Blood pressure and urinary protein were detected. The body length and weight of the pups and the weight of the placenta were recorded. Superoxide dismutase (SOD) activity and levels of malondialdehyde (MDA), placental growth factor (PIGF), and soluble vascular endothelial growth factor receptor-1 (sFIt-1) were measured in the placenta. Pathological changes were observed by hematoxylin-eosin staining. Wnt/β-catenin pathway-related protein expression was detected using Western blot.

**Results:**

Compared with the model group, the PE rats treated with EG had lower blood pressure and urinary protein. The length and weight of the pups and placental weight were increased. Inflammation and necrosis in the placental tissue was improved. SOD level increased, MDA content and sFIt-1/PIGF ratio decreased, and Wnt/β-catenin pathway-related protein expression level increased. Moreover, the results of EG on PE rats increased with higher doses of EG.

**Conclusions:**

EG may activate the Wnt/β-catenin pathway and inhibit oxidative stress, inflammation, and vascular endothelial injury in PE rats, thereby improving the perinatal prognosis of preeclamptic rats. EG may inhibit oxidative stress, inflammation, and vascular endothelial injury through activation of the Wnt/β-catenin pathway in preeclampsia rats, thereby improving perinatal outcomes in PE rats.

## Introduction

Pre-eclampsia (PE) is a common complication of pregnancy with major pathological features including hypertension and proteinuria. The current global prevalence of PE is 2–4%, and the incidence of serious complications is as high as 5–20%^1^, which can lead to maternal organ function damage, intrauterine growth restriction, intrauterine fetal distress, and perinatal death. It seriously affects the life of the mother and child and the subsequent quality of life. There is no effective treatment for PE, and symptoms can only be relieved after delivery of the placenta^2^. It is generally accepted that the main pathogenesis of PE is closely related to oxidative stress, inflammatory factor release, trophoblastic infiltration insufficiency, genetics, and nutrition^3^. Among them, oxidative stress can damage vascular endothelial cell function through reactive oxygen species, which in turn causes activation of the inflammatory system, and the inflammatory response in turn exacerbates endothelial cell function damage^4^^,^ 5.

The Wnt/β-catenin signaling pathway is an important intracellular signaling pathway, in which the activation of upstream molecules such as Wnt1 and Wnt3a can cause intracellular β-catenin accumulation and translocation into the nucleus, which in turn regulates invasion and oxidative stress responses^6^. Activation of the Wnt/β-catenin signaling pathway protects trophoblast cells against hypoxic reoxygenation damage, while inhibition of Wnt/β-catenin pathway causes increased oxygen radical production and oxidative stress damage in trophoblast cells, which contributes to the development of PE^7^^,^ 8.

The main pharmaceutical ingredients of Eucommia granules (EG) include Eucommia and Eucommia leaves, which have the effects of calming the fetus and tonifying the liver and kidney. Both Eucommia and Eucommia leaves have pharmacological effects such as antioxidant and anti-inflammatory, and can be also used to treat secondary hypertension^9^^,^ 10. By modulating biological processes, they regulate hypertension development, including inhibition of inflammation, reduction of oxidative stress levels and regulation of endothelial vasoactive factors^11^^,^ 12. Studies have found that Eucommia is effective in the treatment of hypertensive disorders in pregnancy^13^, but the specific effects and possible mechanisms of EG in PE remain unknown.

Therefore, we investigated the preventive effect of EG on PE rats and the mechanism of EG on PE rats based on Wnt/β-catenin signaling pathway, in order to provide an experimental basis for the clinical prevention of PE.

## Methods

### Animal grouping, modeling, and treatment

Sprague Dawley rats in an estrous cycle were selected and mated in a male to female ratio of 2:1. Vaginal smears were performed daily, and the presence of sperm and vaginal epithelial cells in the smear was considered a successful mating and was recorded as day 1 of pregnancy. Five groups of pregnant rats were randomly assigned (*n* = 6)^14^: control group, model group (PE), low-dose EG group (0.535 g/kg), medium-dose EG group (1.07 g/kg), and high-dose EG group (2.14 g/kg).

The human dose of EG is 10 g/day, or 0.17 g/kg/day. The drug dose conversion ratio for rats and humans is 6.3^15^, so the required granule dose for rats was 1.07 g/kg. Setting 1.07g/kg as the medium dose, the low and high doses would be 0.535 and 2.14 g/kg, respectively.

On the fifth day of pregnancy, the corresponding dose of EG (Guizhou Wonderful Pharmaceutical Co., Ltd., Z52020394, 5 g/bag, dissolve EG in distilled water and dilute to the desired concentration by distilled water) was started to be given by gavage in the EG group, and saline was given to the model group and the control group, which continued until the 21st day of pregnancy.

On day 13 of gestation, rats in all groups except the control group were injected subcutaneously with le-nitroarginine methyl ester (L-NAME).

A dose of 200 mg/kg/d was administered continuously until the 18th day of gestation as a means of establishing a rat model of PE. The control group was given the corresponding dose of saline.

This study was approved and agreed by the Ethics Committee of Gansu Provincial Hospital (No.2023-223).

### Detection of rats (blood pressure, urine protein), littermates (body length, body weight), and placentas (weight)

During the experiment, blood pressure in the caudal artery was measured and recorded on days 9, 15, 18 and 21 of gestation by means of a rat blood pressure meter. Each pregnant rat was placed in a thermostat (40°C) for 15 min to preheat, removed and placed in a fixator, and the systolic blood pressure of the caudal artery was measured using a blood pressure measuring device. The mean value was calculated for three consecutive times. Urine samples were collected from the rats on day 21 in the metabolic cages, and 24-h urine protein was measured using a fully automated biochemical analyzer. At the end of pregnancy, the length and weight of the litter and the weight of the placenta were recorded for each group. The placenta was stored in a -80°C refrigerator and used for subsequent experiments.

### Detection of superoxide dismutase and malondialdehyde levels in placental

The superoxide dismutase (SOD) activity and malondialdehyde (MDA) content of each group of placental tissues were measured according to the kit instructions (Superoxide dismutase assay kit, A001-3-1; Malondialdehyde assay kit, A003-1-1; Nanjing Jiancheng Institute of Biological Engineering, China).

### ELISA for the determination of placental growth factor and soluble vascular endothelial growth factor receptor-1 in placenta

The content of placental growth factor (PIGF) and soluble vascular endothelial growth factor receptor-1 (sFIt-1) in rat placental tissue was measured by enzyme-linked immunosorbent assay (ELISA), according to the kit instructions (Rat PIGF Elisa Kit, ZC-55746; Rat sFlt-1 Elisa Kit, ZC55018; Shanghai Zoucai Biotechnology Co., China).

### Hematoxylin-eosin staining

Rat placental tissues were fixed with 4% paraformaldehyde, dehydrated, transparent, paraffin-embedded, and sectioned. The sections were dried at room temperature. Subsequently, the sections were dewaxed with xylene, hydrated with gradient ethanol solution, rinsed with distilled water. Hematoxylin stain for 5 min, rinsed well with running water, l% hydrochloric acid ethanol fractionation, 1% ammonia wash for 10 s, eosin stain for 30–60 s, rinsed with running water, dehydrated with gradient ethanol: 80% ethanol, 95% ethanol and anhydrous ethanol^16^. Xylene transparent, sections were air-dried, sealed with neutral gum, and histological changes were observed under light microscopy (BA210Digital, McAudi Industries Group Ltd.).

### Western blot

One hundred mg of placental tissue was cut up, added to 1 mL of RIPA lysate, total protein was extracted, and protein was quantified using BCA protein quantification kit [Bioengineering (Shanghai) Co., Ltd., China]. SDS-polyacrylamide gel electrophoresis, polyvinylidene fluoride transfer membrane for 1.5 h, 5% skim milk powder blocking solution for 1 h, and TBST washing membrane were added to the loading buffer, denatured by heating.

β-actin antibody (1:1,000, ab8226), Wnt1 antibody (1:2,000, ab15251), β-catenin antibody (1:4,000, ab32572), c-Myc antibody (1:1,000, ab32072), and Cyclin-D1 antibody (1:1,000, ab16663), respectively, were added, and the membrane was washed with TBST. All the above antibodies were purchased from abcam (Shanghai) Trading Co.

HRP-labeled secondary antibody (1:1,000) was added and incubated at room temperature for 1 h. After PBS washed, luminous solution was added, and ECL chemiluminescence system was used to observe the results.

### Statistical analysis

The data were analyzed with GraphPad Prism 8 and are presented as the means ± standard deviation. Comparisons between the two groups were performed using Student’s t-tests or one-way analysis of variance. Differences with P-values of less than 0.05 were considered statistically significant. P-values of < 0.05 and < 0.01 are indicated with * or # and **or ##, respectively.

## Results

### Effects of Eucommia granules on systolic blood pressure and urinary protein in preeclampsia rats

**Figure 1:**
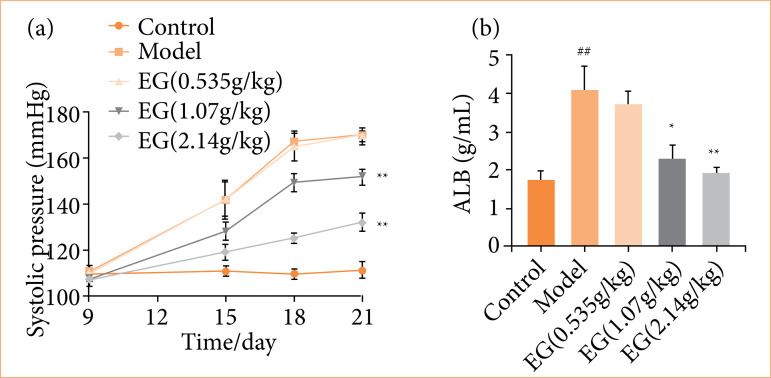
Eucommia granules (EG) reduce systolic blood pressure and 24-h urinary protein in preeclampsia rats. **(a)** Effect of EG on systolic blood pressure in preeclampsia rats. **(b)** Effect of EG on 24-h urinary protein in pre-eclampsia rats. Compared with the control group,

The systolic blood pressure of the rats in each group was measured and recorded at the 9th, 15th, 18th and 21st day of gestation, as shown in Fig. 1a. Among them, there was no significant change in the blood pressure of the control rats during pregnancy. The increase in systolic blood pressure in the model group of preeclamptic rats was the same as that in the low-dose EG group, and the increase in blood pressure in PE rats decreased as the dose of EG increased.

At day 21 of gestation, the systolic blood pressure in both the medium-dose EG group and the high-dose EG group was remarkably lower than that in the model group (*P* < 0.01). Compared with the control rats, the 24-h urine protein was increased in the model group rats at day 21 of gestation (*P* < 0.01).

After treatment with EG, the urinary protein of rats in the medium-dose EG group was lower than that of the model group (P < 0.05), and that of rats in the high-dose EG group was lower than that of the model group (*P* < 0.01) (Fig. 1b). The medium- and high-dose EG could improve hypertension and high urinary protein in pregnant rats with PE.

### Effects of Eucommia granules on the litter and placenta of preeclampsia rats

**Figure 2:**
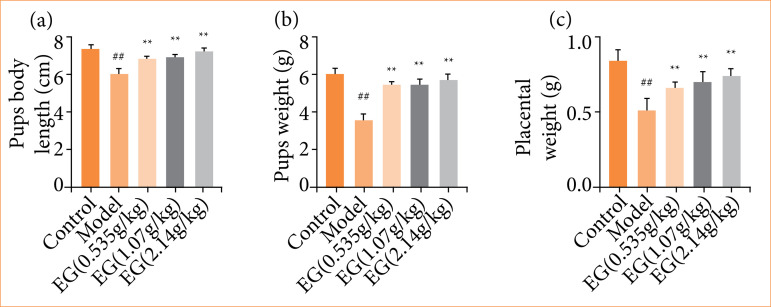
Effect of Eucommia granules (EG) on the litter and placenta of pre-eclampsia rats. **(a)** Effect of EG on the length of litter delivered by pre-eclampsia rats. **(b)** Effect of EG on the weight of litter delivered by pre-eclampsia rats. **(c)** Effect of EG on the weight of placenta delivered by pre-eclampsia rats. Compared with the control group,

The length and weight of the litter and weight of the placenta in each group were recorded at the end of pregnancy. The results are shown in Fig. 2. The length and weight of the litter and weight of the placenta delivered by the pregnant rats in the PE model group were remarkably lower compared with the control rats (P < 0.01). The length and weight of the litter and weight of the placenta delivered by the treated rats increased significantly with the increase of the dose of EG, which were significantly greater than those of the model group in a dose-dependent manner (P < 0.01).

### Effect of Eucommia granules on placental pathology in preeclampsia rats

Morphological changes of placental tissues in each group were observed using HE staining. In the placental tissues of the control rats, no inflammatory cell infiltration was seen, and the thickness of the fetal membrane tissue was uniform with good integrity. There were more immature blood cells in the labyrinthine trophoblast and villi stem cells, and the cytoplasm was more eosinophilic.

In the placental tissue of the model group, the placental tissue was structurally intact. However, the trophoblast cells were degenerated and necrotic, with varying degrees of inflammatory cell infiltration. Nuclei disintegration and cytoplasmic lysis were also clearly observed. At the same time, eosinophilia was enhanced and accompanied by more hemorrhage.

In the placental tissues of rats in the EG low-dose group and EG medium-dose group, the degenerative necrosis of trophoblast cells was slightly reduced, the inflammatory cell infiltration was reduced, the nuclei were fragmented, accompanied by hemorrhage, and dilated blood sinuses, and no other pathological changes were observed.

**Figure 3:**
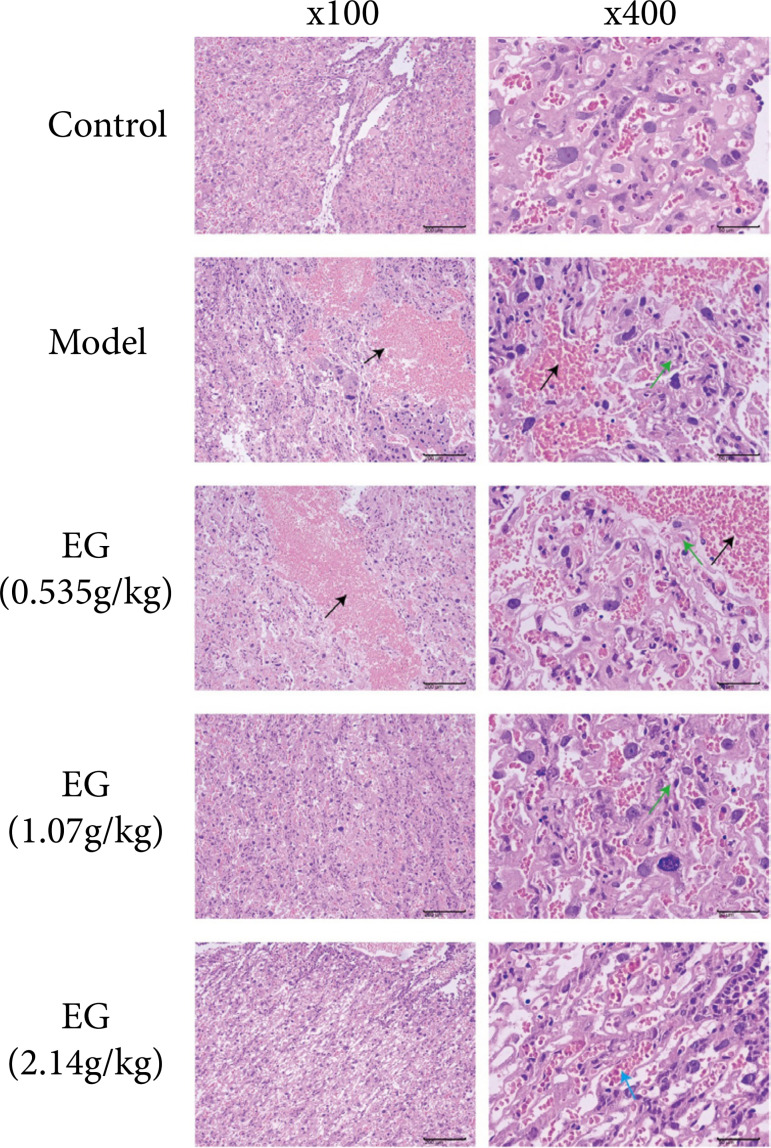
Effect of Eucommia granules (EG) on placental pathology in pre-eclampsia rats (black arrow indicates hemorrhage, green arrow indicates degenerative necrosis of trophoblast cells, blue arrow indicates dilated blood sinus).

Source: Elaborated by the authors.

In the placental tissue of rats in the EG high-dose group, no cell degeneration necrosis, inflammatory cell infiltration and hemorrhage were observed, there was blood sinus dilation, and no other pathological changes were seen (Fig. 3).

### Effect of Eucommia granules on antioxidant indexes in preeclampsia rats

**Figure 4:**
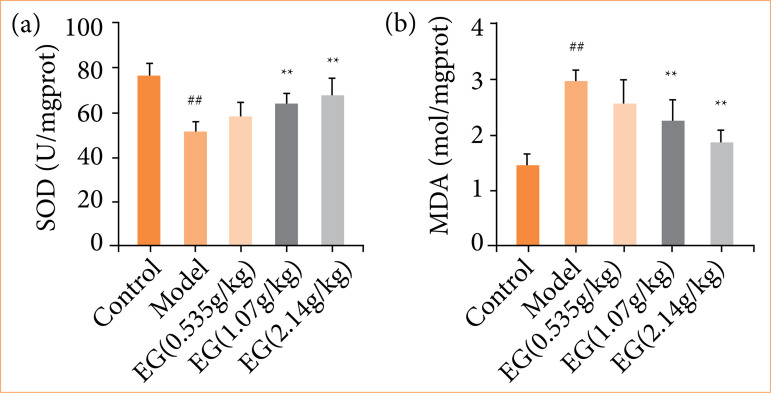
Eucommia granules (EG) improve oxidative damage in the placenta of pre-eclampsia rats. **(a)** Effect of EG on SOD activity of placental tissues. **(b)** Effect of EG on MDA content of placental tissues. Compared with the control group,

Oxidative stress has a close relationship with the pathogenesis of PE. Therefore, SOD activity and MDA content were tested in the placental tissue of experimental rats. The results showed that SOD activity was remarkably lower (*P* < 0.01), and MDA content was higher (*P* < 0.01) in the model group compared with the control group. Compared with the model group, the changes of SOD activity and MDA content in the low-dose EG group were not significant. The SOD activity in the medium-dose EG group and the high-dose EG group was significantly increased (*P* < 0.01), and the MDA content was significantly reduced (*P* < 0.01). The medium-dose and high-dose EG could improve the oxidative stress injury in PE rats (Fig. 4).

### Effect of Eucommia granules on soluble vascular endothelial growth factor receptor-1/placental growth factor in preeclampsia rats

PIGF, a pro-angiogenic factor, interacts with the anti-angiogenic factor sFIt-1 to affect the placental vascular spiral artery shaping process, which in turn affects placental perfusion and promotes the development of PE^17^. The level of sFIt-1 and PIGF and the size of the sFIt-1/PIGF ratio have differential diagnostic implications for PE^18^^,^ 19. The levels of PIGF and sFlt-1 in placental tissue were tested using ELISA.

Placental tissues of rats in the model group contained remarkably less PIGF than those in the control group (*P* < 0.01), while the content of sFIt-1 was increased (*P* < 0.01), and the size of sFIt-1/PIGF ratio was significantly increased. The low-dose EG group did not show any significant differences in the levels of sFIt-1 and PIGF compared with the model group.

As the dose of EG increased, in the placental tissues of the middle-dose EG group, the PIGF content was greater than that of the model group (*P* < 0.05), the sFIt-1 content was lower than that of the model group (*P* < 0.01), and the size of the sFIt-1/PIGF ratio was significantly lower (*P* < 0.01).

**Figure 5:**
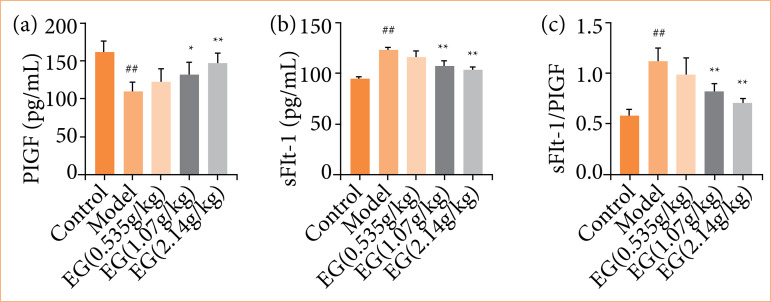
Eucommia granules (EG) reduce sFIt-1/PIGF in pre-eclampsia rats. **(a)** Effect of EG on placental tissue PIGF content. **(b)** Effect of EG on placental tissue sFIt-1 content. **(c)** Effect of EG on sFIt-1/PIGF. Compared with the control group,

In the placental tissue of the high-dose EG group, as compared to the model group, the PIGF content was significantly higher (*P* < 0.01). The sFIt-1 content and sFIt-1/PIGF ratio size were remarkably smaller than those of the model group (*P* < 0.01). EG treatment effectively improved endothelial injury in PE rats (Fig. 5).

### Effect of Eucommia granules on placenta of preeclampsia rats Wnt/β-catenin pathway

To investigate the effect of EG on the Wnt/β-catenin pathway in the placenta of PE rats, the expression levels of Wnt1, β-catenin, c-Myc and Cyclin-D1 proteins in placental tissues were measured by Western blot.

**Figure 6:**
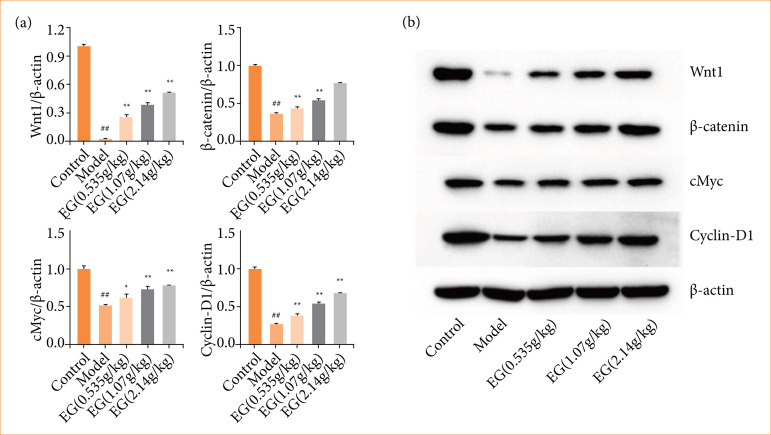
Upregulation of placental Wnt/β-catenin pathway in pre-eclampsia rats by Eucommia granules (EG). **(a)** Statistics of Wnt1, β-catenin, c-Myc and Cycli n-D1 protein expression levels in placental tissues of rats in each group. **(b)** Results of Western blotting assay of placental Wnt/β-catenin pathway protein expression levels. Compared with the control group,

The results showed (Fig. 6) that the expression levels of Wnt1, β-catenin, c-Myc and Cyclin-D1 proteins in the placental tissues of the model group were lower (*P* < 0.01) when compared with the control group. The activity of Wnt/β-catenin pathway was inhibited in the placental tissues of PE rats. Compared with the model group, Wnt1, β-catenin, c-Myc and Cyclin-D1 protein expression levels were significantly upregulated in the placental tissues of rats treated with EG (P < 0.05), and the higher the dose of EG, the higher the protein expression level. EG up-regulated Wnt/β-catenin pathway activity in PE rats.

## Discussion

PE is a major cause of high maternal and perinatal morbidity and mortality^20^. Currently, it can only be treated by symptomatic treatment combined with termination of pregnancy^21^, and it would be of great clinical significance if PE could be prevented or treated from the perspective of pathogenesis.

In this study, we used different doses of EG to treat PE rats and observed that higher doses of EG could reduce blood pressure and 24-h urine protein in PE rats, increase the weight of the litter and placenta, and improve the inflammatory cell infiltration and degenerative necrosis of placental tissue. It indicates that EG can improve the perinatal outcome and reduce the placental pathological damage in PE rats. Therefore, we continued to investigate the possible mechanism of the action of EG.

Although the etiology of PE is still controversial, it is generally accepted that hypoxia and oxidative stress caused by placental insufficiency play an important role in the development of the disease^22^. The literature suggests that suppression of oxidative stress is effective in reducing the incidence of PE in high-risk pregnant women^23^.

In addition, studies have shown that maternal factors such as genetic or immune disorders cause placental vascular remodeling disorders and insufficient infiltration of trophoblast cells into the uterine myometrium, resulting in reduced placental perfusion, and thus localized placental tissues are in a state of ischemia and hypoxia, which further causes the onset and progression of PE^3^^,^ 24. Peripheral blood oxidative stress indicators such as MDA levels and ROS in patients with pregnancy-induced hypertension and eclampsia showed a positive correlation with the degree of endothelial cell damage as blood pressure increased. On the contrary, SOD, as a major antioxidant compound, can be seen to be significantly reduced in endothelial cell-damaging diseases^25^.

In this study, the MDA level in the placental tissues was lower than that of the model group, while the SOD level was higher than that of the model group in PE rats treated with medium- and high-dose EG, suggesting that EG can effectively improve the oxidative stress in placental tissues of PE rats.

PIGF is an important component of vascular endothelial growth factor, which can promote the proliferation and migration of vascular endothelial growth factor in the body and local angiogenesis^26^. In addition, PIGF is one of the important proteins in pregnancy, which promotes the production of placental vascular network and ensures fetal growth and development^27^.

sFIt-1 is an anti-vascular growth factor, which can cause vascular endothelial injury and angiogenesis and reflect the level of vascular inflammation^28^. It is expressed in high concentrations on human placental villi and trophoblast cells, and its main biological functions include: downregulating and inhibiting the biological function of PIGF and causing impaired angiogenesis^28^.

In our study, the PIGF content in placental tissues was increased after medium- and high-dose EG treatment, while the sFIt-1 content and sFIt-1/PIGF ratio were remarkably reduced. Combined with the HE staining results, it was observed that the intervention of EG could improve inflammation and vascular endothelial damage and promote angiogenesis in preeclampsia rats.

The oxidative stress response is an important biological link regulated by the Wnt/β-catenin signaling pathway^29^. Studies have demonstrated the Wnt/β-catenin pathway is involved in angiogenesis under ischemia and hypoxia and that enhancement of the Wnt/β-catenin pathway is beneficial in the promotion of angiogenesis^30^. The canonical Wnt/β-catenin pathway is triggered when the Wnts are combined with Wnt ligands (e.g., Wnt1). The expression of Wnt1 is higher in trophoblast cells of early pregnancy than in trophoblast cells of late pregnancy, indicating that Wnt1 can regulate trophoblast invasion^31^. β-catenin is a key factor in the Wnt/β-catenin pathway. Reducing β-catenin levels can lead to a decrease in the invasive capacity of human choroidal trophoblast cells^32^.

In the presence of Wnt ligands, the Wnt signaling component binds to the membrane receptor Frizzled, leading to an increase in β-catenin, which in turn activates the downstream target genes cyclinD1 and c-Myc^33^.

Wang’s study found that mRNA expression of Wnt1, β-catenin, c-myc and cyclinD1 was reduced in PE placental tissue and that the Wnt/β-catenin pathway may play an important role in the pathogenesis of PE by regulating trophoblast invasion and proliferation^34^. In the current study, the expression levels of Wnt1, β-catenin, c-Myc, and Cyclin-D1 were remarkably reduced in placental tissues of PE rats, while the expression of Wnt/β-catenin pathway-related proteins in placental tissues was higher than that in the model group after medium- or high-dose EG treatment. This suggests that the effect of EG on PE rats may be related to the Wnt/β-catenin pathway.

## Conclusion

This study demonstrated that higher doses of EG improved perinatal outcomes and pathological damage in PE rats, inhibited oxidative stress, inflammation, and endothelial damage phenomena, and activated the Wnt/β-catenin pathway related. Based on the present study, we should further verify the mechanism of action of EG related to Wnt/β-catenin by adding pathway inhibitors to the PE rats for intervention, which will be the focus of our next research.

## Data Availability

The data will be available upon request.
